# Different Forms of Tumor Vascularization and Their Clinical Implications Focusing on Vessel Co-option in Colorectal Cancer Liver Metastases

**DOI:** 10.3389/fcell.2021.612774

**Published:** 2021-04-12

**Authors:** Gwendolyn Haas, Shuang Fan, Michael Ghadimi, Tiago De Oliveira, Lena-Christin Conradi

**Affiliations:** Department of General, Visceral and Pediatric Surgery, University Medical Center Göttingen, Göttingen, Germany

**Keywords:** angiogenesis, colorectal cancer, vessel co-option, liver metastasases, histopathological growth patterns, lung metastasis, brain metastasis

## Abstract

In modern anti-cancer therapy of metastatic colorectal cancer (mCRC) the anti-angiogenic treatment targeting sprouting angiogenesis is firmly established for more than a decade. However, its clinical benefits still remain limited. As liver metastases (LM) represent the most common metastatic site of colorectal cancer and affect approximately one-quarter of the patients diagnosed with this malignancy, its treatment is an essential aspect for patients' prognosis. Especially in the perioperative setting, the application of anti-angiogenic drugs represents a therapeutic option that may be used in case of high-risk or borderline resectable colorectal cancer liver metastases (CRCLM) in order to achieve secondary resectability. Regarding CRCLM, one reason for the limitations of anti-angiogenic treatment may be represented by vessel co-option (VCO), which is an alternative mechanism of blood supply that differs fundamentally from the well-known sprouting angiogenesis and occurs in a significant fraction of CRCLM. In this scenario, tumor cells hijack pre-existing mature vessels of the host organ independently from stimulating new vessels formation. This represents an escape mechanism from common anti-angiogenic anti-cancer treatments, as they primarily target the main trigger of sprouting angiogenesis, the vascular endothelial growth factor A. Moreover, the mechanism of blood supply in CRCLM can be deduced from their phenotypic histopathological growth pattern (HGP). For that, a specific guideline has already been implemented. These HGP vary not only regarding their blood supply, but also concerning their tumor microenvironment (TME), as notable differences in immune cell infiltration and desmoplastic reaction surrounding the CRCLM can be observed. The latter actually serves as one of the central criteria for the classification of the HGP. Regarding the clinically relevant effects of the HGP, it is still a topic of research whether the VCO-subgroup of CRCLM results in an impaired treatment response to anti-angiogenic treatment when compared to an angiogenic subgroup. However, it is well-proved, that VCO in CRCLM generally relates to an inferior survival compared to the angiogenic subgroup. Altogether the different types of blood supply result in a relevant influence on the patients' prognosis. This reinforces the need of an extended understanding of the underlying mechanisms of VCO in CRCLM with the aim to generate more comprehensive approaches which can target tumor vessels alternatively or even other components of the TME. This review aims to augment the current state of knowledge on VCO in CRCLM and other tumor entities and its impact on anti-angiogenic anti-cancer therapy.

## Role of Tumor Vessels and Associated Resistance Mechanisms

Tumor vessels can be very heterogeneous in their characteristics and mode of formation, depending on the tumor entity and its host tissue. The tumor vasculature is a well-established therapeutic target in addition to classical anti-tumor systemic therapy approaches. For targeting the tumor vasculature as an anti-cancer treatment, the most common modes of blood vessel development should be considered and will be described in this section.

### Sprouting Angiogenesis

**Figure 1:**
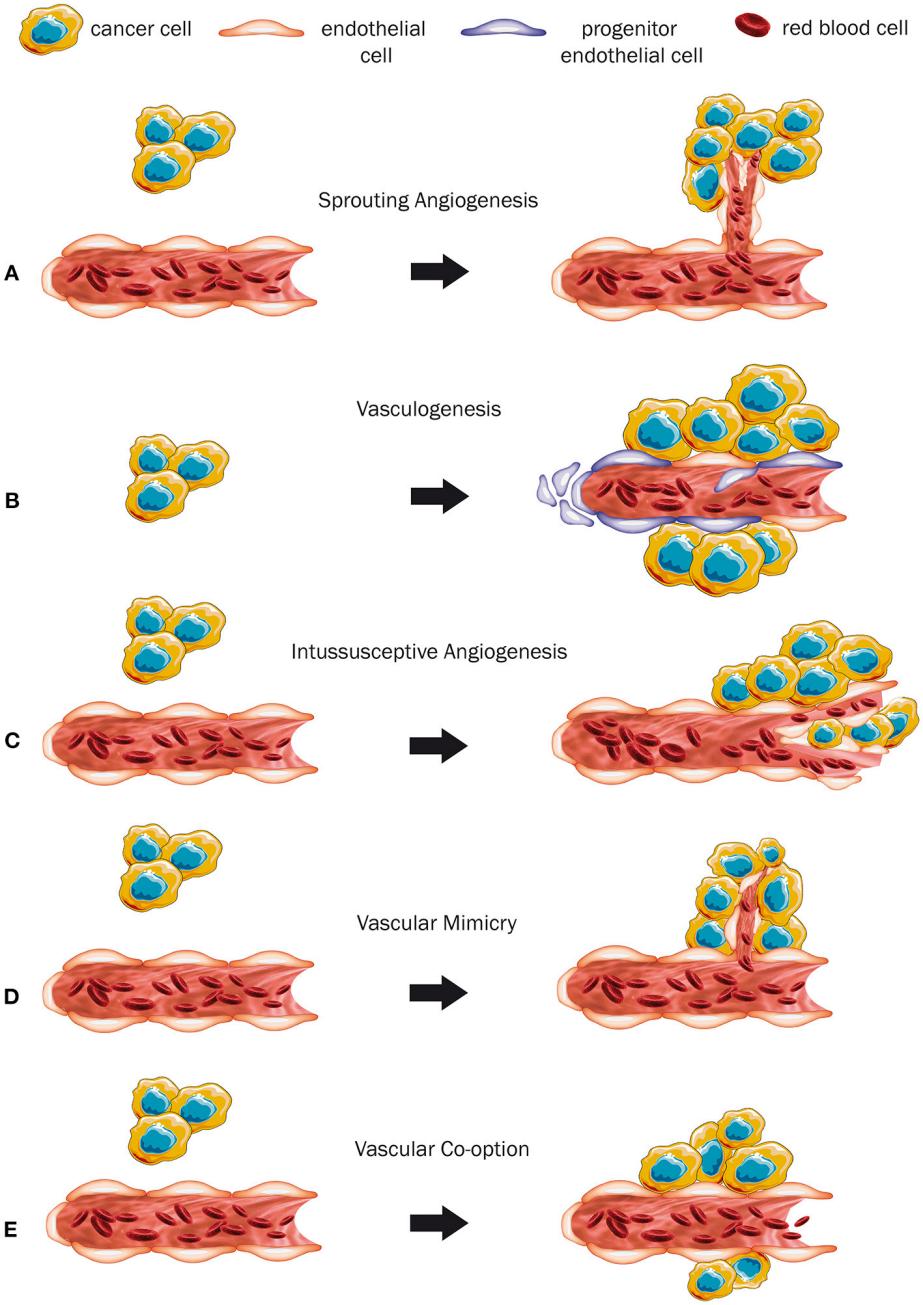
Modes of vascularization **(A–E)**. **(A)** The mechanism of sprouting angiogenesis. As the tumor grows, sprouting of new immature vessels from a mature preexisting vessel is induced. **(B)** Vasculogenesis, a mechanism where a completely new vessel is formed by progenitor cells. **(C)** Intussusceptive angiogenesis. Hereby, a pre-existing vessel is promoted to split itself into two new ones. **(D)** Vascular mimicry. The tumor cells form a vessel-like structure themselves. **(E)** Co-option of a pre-existing mature blood vessel by expanding tumor cells.

Sprouting angiogenesis is presumably the best characterized way of how tumors secure their blood and nutrients supply. Basically, this mechanism comprises the proliferation and migration of endothelial cells for generating a new immature vessel growing from a mature one (Hanahan and Folkman, 1996; Figure 1A). Thereby, the basal lamina of the mature original vessel becomes discontinuous, evolves various layers, or even completely fades. Next, endothelial cells and pericytes start proliferating, accompanied by endothelial cell migration (Paku and Paweletz, 1991). The key player driving this process is vascular endothelial growth factor (VEGF), which promotes sprouting angiogenesis in physiological situations during development and growth of normal tissues (e.g., muscles) as well as malignant tumors (Melincovici et al., 2018).

### Vasculogenesis

In the event of vasculogenesis, the release of cytokines and chemokines recruits endothelial and hematopoietic progenitor cells from the circulating blood stream to help generate new vessels (Rafii et al., 2008; Figure 1B). There is evidence that the endothelial progenitor cells can also express different growth factors and probably promote simultaneously sprouting angiogenesis (Urbich and Dimmeler, 2004). The mechanism of vasculogenesis is well-known to take place during embryonic development (Risau and Flamme, 1995), wound healing, after ischemic events, and for tumor growth (Rafii et al., 2008).

### Intussusceptive Angiogenesis

During intussusceptive or non-sprouting angiogenesis, new blood vessels are generated by splitting and remodeling of pre-existing ones (Patan et al., 1996; Burri et al., 2004; Mentzer and Konerding, 2014). Its hallmark is the formation of intraluminal pillars at the beginning of the process, which will be further invaded by pericytes and split up giving rise to two new capillaries (Karthik et al., 2018; Figure 1C). This mechanism provides an enlargement of the vessel surface, which improves gas and nutrient exchange of the pre-existing vasculature (Burri et al., 2004). Factors like hemodynamic conditions, oxygen levels, and pro-angiogenic molecules are being discussed to contribute to its initiation (Djonov and Makanya, 2005). Earlier studies suggested that angiopoetins I and II, platelet-derived growth factor beta polypeptide, and the fibroblast growth factor contribute to the regulation of intussusceptive angiogenesis (De Spiegelaere et al., 2012). Interestingly, VEGF appears to have a subordinated role in this process and its accurate contribution to intussusceptive angiogenesis is still not fully understood (De Spiegelaere et al., 2012). Nevertheless, intussusceptive angiogenesis can be considered a “complementary method” to sprouting angiogenesis (Karthik et al., 2018) and has been described to occur physiologically in some organs, during tissue repair and in several tumors (Hillen and Griffioen, 2007; Ribatti and Djonov, 2012).

### Vascular Mimicry

Vascular mimicry describes the capacity of cancer cells to form structures that are similar to regular vessels by themselves, independently from common endothelial cells (Figure 1D). In this scenario the tumor cells even adopt endothelial features (Maniotis et al., 1999). Some of its molecular drivers, such as vascular endothelial cadherin, VEGF-receptor 1, metalloproteases, hypoxia-inducible factor 1α, and others have already been identified (Qiao et al., 2015), but the exact underlying mechanism for the conversion of cancer cells into endothelial-like cells still needs to be investigated further (Qian et al., 2016). The phenomenon of vascular mimicry occurs in a variety of tumor entities, including melanoma, osteosarcoma, Ewing sarcoma, ovarian cancer, breast cancer, prostate cancer, lung cancer, head and neck tumors, malignancies of the gastrointestinal tract, e.g., esophageal, gastric, hepatocellular, and colorectal cancer, malignancies of the gallbladder, and in several intracranial tumors, such as glioblastoma, astrocytoma, and non-functioning pituitary adenoma (Qiao et al., 2015; Ge and Luo, 2018).

### Vessel Co-option

Vessel co-option (VCO) represents an entirely different mechanism of acquiring blood supply without the need of generating new vessels. Instead, tumor cells hijack pre-existing mature vessels of the host organ (Latacz et al., 2020a; Figure 1E). This phenomenon is mostly observed in highly vascularized organs like the brain, the lungs, and the liver, which provide an opulent supply of nutrients and oxygen (Donnem et al., 2013). The identification of VCO is often feasible through common light microscopy and basic histological analysis (Donnem et al., 2013). Vessel co-opting tumors tend to grow less destructive (Pezzella et al., 1997), can show a close contact to the surrounding tissue (Vermeulen et al., 2001), and grow in an infiltrative manner (van Dam et al., 2017; Kuczynski et al., 2019). Kuczynski et al. (2019) conflated histopathological key characteristics of VCO. As tumors grow along the pre-existing vessels, they tend to mimic the histological morphology of the host organ, rather than destroying the surrounding tissue (van Dam et al., 2017; Kuczynski et al., 2019). Also, the morphology and structure of the co-opted vessels might remain unaltered to some extent. However, this latter aspect is a relatively frail criterion, as vessel alterations can occur nevertheless (Kuczynski et al., 2019). In some malignancies, e.g., CRCLM and lung metastases, VCO is directly related to specific growth patterns of the tumor, so that the occurrence of VCO can be simply deduced from morphological aspects of the tumor (Pezzella et al., 1997; Bridgeman et al., 2017; van Dam et al., 2017). Immunohistochemical analysis allows the differentiation between mature and immature vessels, which helps to distinguish between VCO and sprouting angiogenesis. The observed level of smooth muscle actin, which serves as a pericyte marker, can indicate the pericyte coverage of the vessels (Donnem et al., 2013; Lazaris et al., 2018). Immuno-co-staining for endothelial and proliferation markers, such as CD34 and Ki67, can be used to identify proliferating vessels (Lazaris et al., 2018). Thus, a considerable layer of pericytes and the lack of proliferating cells can indicate the presence of VCO. Additionally, an increased micro-vessel density is often used as a supportive criterion to describe the presence of angiogenesis, and is often misunderstood as a reliable indicator for the absence of VCO (Kuczynski et al., 2019). Conversely, a level of microvascular density which resembles the one in the surrounding tissue can indirectly indicate the absence of angiogenesis (Donnem et al., 2013). Nevertheless, also increased levels of microvascular density were observed in vessel co-opting tumors (Lazaris et al., 2018), reinforcing that the micro-vessel density is an inconsistent criterion, and therefore it should be used as a supportive element, rather than as a defining one (Donnem et al., 2013; Kuczynski et al., 2019).

A related mechanism used by cancer cells to benefit from pre-existing, but also from newly formed vessels, is the process of pericytic mimicry (PM) (Lugassy et al., 2020). The histopathological counterpart of the mechanism of this phenomenon is angiotropism (Lugassy et al., 2020), which is characterized by the localization of the cancer cells in a pericytic location, migrating along the abluminal side of vessels (Barnhill and Lugassy, 2004; Lugassy et al., 2020). This spatial proximity to vessels naturally blends PM to VCO (Lugassy et al., 2020). Although PM represents the migratory process, whereas vessel co-opting cells are more invading (Lugassy et al., 2020), there is evidence that both processes are at least closely interlinked or might even be identical (Bentolila et al., 2016; Barnhill et al., 2018). In a murine model it was shown that the implantation of melanoma cells in the brain resulted in melanoma cells spreading on the abluminal surface of pre-existing vessels (Bentolila et al., 2016). Thus, characteristics of VCO and PM were found coincidentally (Bentolila et al., 2016). However, in the event of VCO, an intravascular metastatic process might be commonly expected (Bentolila et al., 2016). With regard to the exclusively extraluminal migration process of PM (Lugassy et al., 2020), and considering it as an alternative way of metastasis formation (Bentolila et al., 2016), it would be highly interesting to investigate further whether metastases that are known for VCO, that are known for VCO, e.g., vessel co-opting CRCLM, reached the side, reached the side of metastases via an intravascular or an extravascular route.

So far, the exact underlying mechanisms of VCO are still not fully understood. To date, despite the previously described tumor cell invasion, other variables such as cell adhesion mechanisms seem to play a role in VCO (Kuczynski et al., 2019). In brain metastases, it was shown that the L1 cell adhesion molecule (L1CAM), an axon pathfinding molecule, is involved in the process of VCO (Valiente et al., 2014). Previous analyses, obtained through a murine model for brain metastases, also pointed out the crucial role of cell adhesion in VCO and showed that the contractile cytoskeleton of the tumor cells contributes to VCO establishment (Dome et al., 2003). Other cell adhesion molecules such as, β1-integrin and α3-integrin have also been associated with VCO in brain metastases *in vivo* (Carbonell et al., 2009; Bugyik et al., 2011). Following this rationale, Frentzas et al. (2016) were able to prove *in vivo* that the knockdown of actin-related protein complex 2/3, which is involved in cancer cell motility and invasion (Otsubo et al., 2004), has the potential to suppress VCO (Frentzas et al., 2016). Therefore, the currently available data suggest that cancer cell motility might also directly contribute to VCO. Recently, comparing mainly angiogenic CRCLM with mainly vessel co-opting CRCLM, a higher expression of lysyl oxidase-like 4 protein (LOXL4) was observed in neutrophils near the vessel co-opting CRCLM (Palmieri et al., 2020). Lysyl oxidases are physiologically involved in crosslinking of collagen and elastin (Lucero and Kagan, 2006), but in tumors, they are involved in malignant processes, such as epithelial-to-mesenchymal transition and promotion of metastases through remodeling of the tumor microenvironment (TME) (Xiao and Ge, 2012). This association of a high expression of LOXL4 in neutrophils near vessel co-opting CRCLM in contrast to a lower expression of LOXL4 in neutrophils close to angiogenic CRCLM might emphasize the importance of the interplay between TME and tumor cells.

#### Resistance to Anti-angiogenic Treatment

Since the Food and Drug Administration (FDA) approved bevacizumab for approval of bevacizumab for metastatic colorectal cancer (mCRC) in 2004 (STN-125085/0), the anti-angiogenic approach in anti-cancer therapies has been well-established and further extended (Garcia et al., 2020). As angiogenesis represents one of the hallmarks of cancer (Hanahan and Weinberg, 2000), the anti-angiogenic treatment was expected to show an undeniable efficacy in anti-cancer treatment. Knowing the different kinds of blood supply, it remains obvious that targeting sprouting angiogenesis cannot inhibit all types of tumor growth patterns. In experimental settings it has been shown that vascular mimicry seems to resist anti-angiogenic agents, such as anginex, TNP-470, endostatin, bevacizumab, and vatanib (van der Schaft et al., 2004; Angara et al., 2018). Moreover, there are indications that further tumor progression after inhibition of angiogenesis might be linked to the formation of vascular mimicry (Xu et al., 2012).

Recently, *in vitro* data highlighted the association of tumor stiffness with an impaired response to anti-angiogenic treatment in CRCLM, whereas softer tumor tissues seem to respond better to anti-angiogenic treatment (Shen et al., 2020). The key players of this process in CRCLM are metastases-associated fibroblasts (Shen et al., 2020), and therefore matrix-related components of the CRCLM TME. This indicates an important role of TME alterations in CRCLM on clinical effects of anti-angiogenic treatment. Moreover, VCO represents a way of blood supply, which should be unaffected by anti-angiogenic anti-cancer treatment approaches (Bergers and Hanahan, 2008). Various studies have already confirmed this hypothesis by showing that VCO serves as resistance mechanism to anti-angiogenic agents in several cancer entities, such as glioblastoma, cerebral melanoma metastases, hepatocellular carcinoma, lung metastases, and CRCLM (Rubenstein et al., 2000; Leenders et al., 2004; Keunen et al., 2011; Frentzas et al., 2016; Kuczynski et al., 2016; Bridgeman et al., 2017). In a recent review, Kuczynski and Reynolds (2020) comprehensively summarize the role of VCO as a resistance mechanism to anti-angiogenic therapy. Based on the available current knowledge, the development of new experimental models (*in vitro, in vivo*, or mathematical) and possible clinical therapeutic guidelines, such as the ones suggested by Voutouri et al. (2019), are imperative to a better understanding of anti-angiogenic therapy and its relationship with different types of blood supply.

## The Tumors' Border Defines the Histopathological Growth Pattern

As previously mentioned, in many tumor entities the occurrence of VCO can be deduced from the evaluation of its morphologic criteria (Kuczynski et al., 2019). In CRCLM the mode of blood supply used by the cancer cells comes in association with a specific morphological appearance. A pivotal aspect consists in the border of the CRCLM and its relation to the surrounding liver tissue, when analyzed under the light microscope by haematoxylin and eosin stainings (van Dam et al., 2017). These morphologic characteristics are classified as HGP, which, which can be understood as a clearly differing TME between the different HGP. In human CRCLM, three main HGP are observed and well-defined according to the international consensus guideline (van Dam et al., 2017).

### Desmoplastic HGP

**Figure 2:**
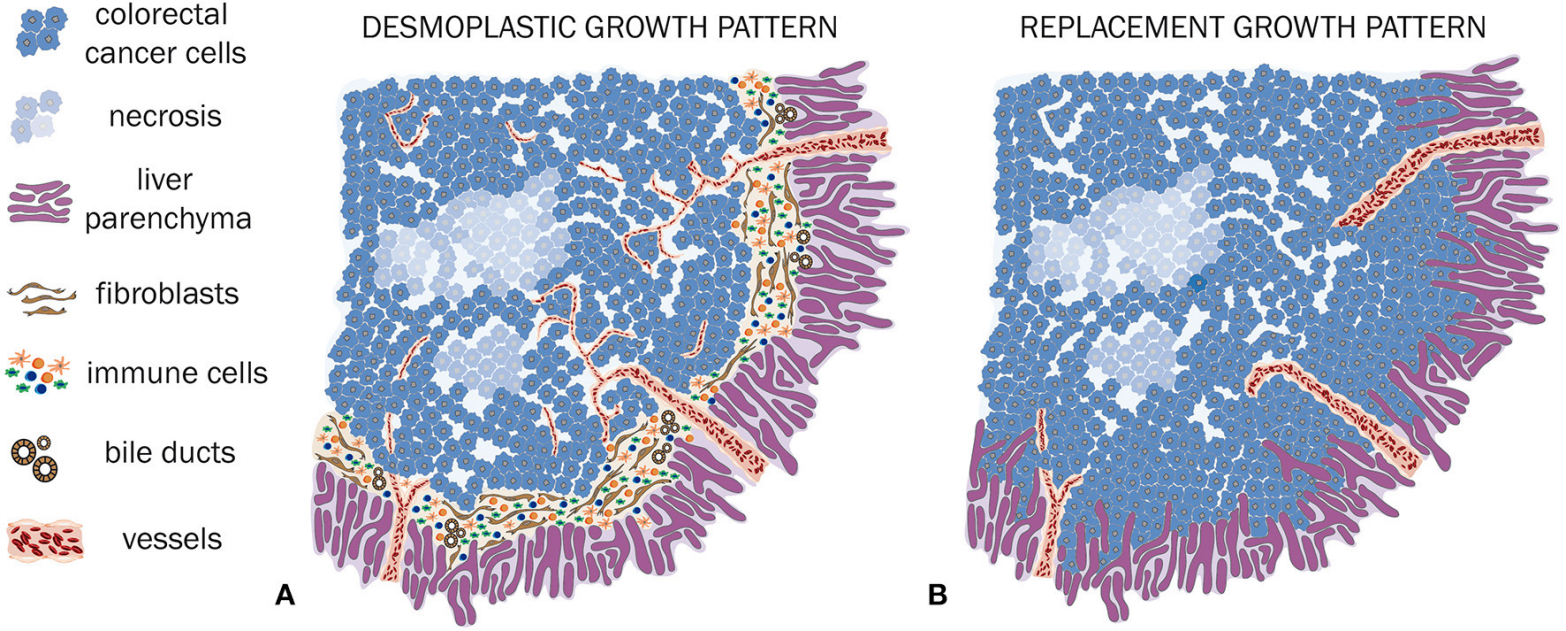
HGP of CRCLM **(A,B)**. **(A)** Schematic illustration of a desmoplastic CRCLM. Hereby, the defining desmoplastic rim between tumor and liver tissue is clearly visible. A dense immune cell infiltration is surrounding and also invading the metastasis. Within the desmoplastic rim, the ductular reaction is illustrated. The ingrowing vessels demonstrate sprouting angiogenesis, which is characteristic for desmoplastic CRCLM. **(B)** Schematic illustration of replacement CRCLM. This type of metastases is characterized by tumor cells, mimicking the liver architecture, co-option of mature pre-existing vessels, and absence of an inflammatory and fibroblastic rim.

**Figure 3:**
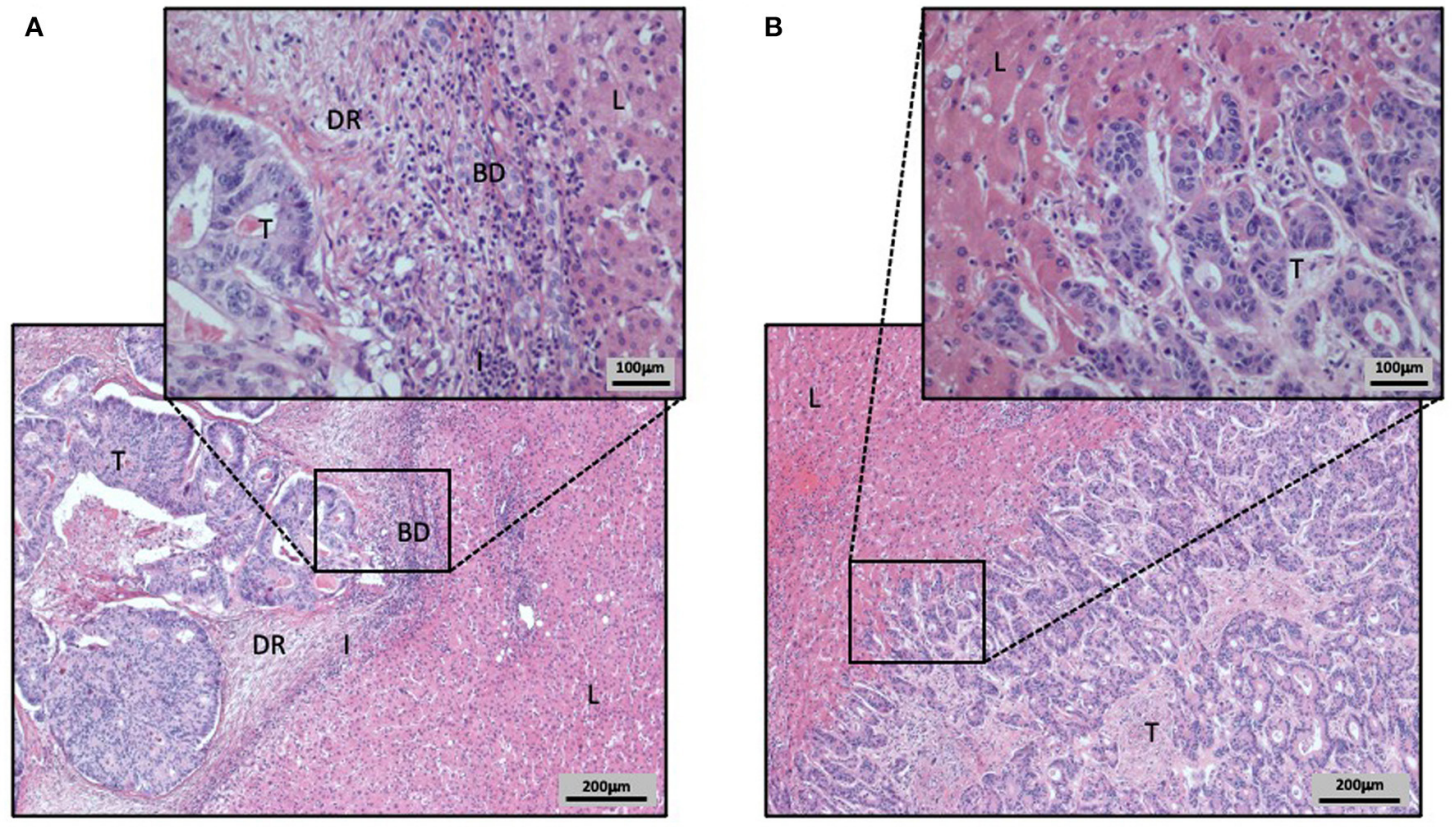
Hematoxylin and Eosin stained CRCLM with different HGP **(A,B)**. Liver tissue is labeled as L, tumor tissue as T, ductular reaction of bile ducts as BD, the immune cell infiltration as I, and the desmoplastic rim as DR. **(A)** CRCLM with desmoplastic HGP. The CRCLM is differentiated from the surrounding liver tissue by the HGP defining desmoplastic rim. Additionally, in the desmoplastic rim the ductular reaction, composed by proliferating bile ducts, and the inflammatory infiltration are visible. **(B)** CRCLM with replacement HGP. The close cell–cell contact of tumor cells and hepatocytes is clearly visible, as the tumor cells invade the liver cell plates.

In case of a desmoplastic HGP, the CRCLM tissue is surrounded by a rim, composed frequently by fibroblasts and immune cells, which are generally lymphocytes. Also, a ductular reaction, due to increased bile ducts proliferation, can be observed (Vermeulen et al., 2001; Nielsen et al., 2014; van Dam et al., 2017). The desmoplastic rim is the main feature of this HGP, preventing direct contact between tumor cells and the adjacent hepatocytes (Vermeulen et al., 2001; van Dam et al., 2017; Figures 2A, 3A). Of crucial relevance, different independent studies already showed that desmoplastic CRCLM ensure their blood supply via sprouting angiogenesis (Vermeulen et al., 2001; Stessels et al., 2004; Lazaris et al., 2018).

### Replacement HGP

The replacement HGP differentiates sharply from the previously described desmoplastic HGP. In this type of HGP, when expanding, tumor cells grow within the liver cell plates while replacing the pre-existing hepatocytes (Vermeulen et al., 2001; Stessels et al., 2004; van Dam et al., 2017). Thus, a close cell–cell contact between tumor cells and hepatocytes is included in the definition of the replacement HGP (van Dam et al., 2017). A key consequence of this HGP consists in the utilization of VCO to ensure their blood supply. Hereby, VCO represents the predominant way of blood supply, as the sinusoidal vessels are co-opted by the tumor cells (Vermeulen et al., 2001; Stessels et al., 2004; Frentzas et al., 2016; van Dam et al., 2017; Figures 2B, 3B).

### Pushing HGP

The pushing HGP is characterized by the CRCLM pushing away the surrounding normal tissue (Vermeulen et al., 2001; Stessels et al., 2004; van Dam et al., 2017). Although there is explicitly no spatial segregation between CRCLM and liver tissue, there is no direct contact between the CRCLM cells and the hepatocytes (van Dam et al., 2017). In spite of this lack of a boundary, the tumor cells do not infiltrate the liver tissue (van Dam et al., 2017). Just like in the desmoplastic HGP, the CRCLM with pushing HGP also obtain blood and nutrients via sprouting angiogenesis (Vermeulen et al., 2001; Eefsen et al., 2012; Van den Eynden et al., 2013; van Dam et al., 2017).

### The Tumor Microenvironment of HGP Is Characterized by Different Levels of Immune Cell Infiltration

For almost two decades, the differences in immune cell infiltration between different HGP is recognized as an important feature which is further studied in current research (Vermeulen et al., 2001). Generally, among the common HGP the immune cell infiltration is most dense in desmoplastic CRCLM, followed by pushing CRCLM and lastly by replacement CRCLM (Vermeulen et al., 2001; Nielsen et al., 2014; van Dam et al., 2017).

Since 2017, when the definition of different immune phenotypes of tumors was suggested, tumors can be classified according to their immune infiltration level as either an (i) immune-desert type, an (ii) immune-excluded type, or an (iii) inflamed type (Chen and Mellman, 2017).

Stremitzer et al. (2020) were able to show a significant association of the desmoplastic HGP with the inflamed immune phenotype, in a group of patients with CRCLM that were preoperatively treated with chemotherapy and bevacizumab. This inflamed immune phenotype was defined by the presence of a considerable infiltration of CD8 positive immune cells (Stremitzer et al., 2020; Figures 2A, 3A). In contrast to the desmoplastic HGP, the replacement HGP shows a far reduced association with CD8 positive immune cells, when comparing those two HGP within one mixed metastasis that displayed both, replacement and desmoplastic growth (van Dam et al., 2018). Thus, the replacement HGP often tends to exhibit the desert type of immune phenotype (van Dam et al., 2018; Figures 2B, 3B).

Interestingly, in CRCLM that mainly express the vessel co-opting replacement HGP, a higher level of LOXL4-positive neutrophils has been observed in their TME when compared to angiogenic, desmoplastic HGP (Palmieri et al., 2020). Nevertheless, also in CRCLM with a predominantly desmoplastic HGP, LOXL4-positive neutrophils can be observed, but interestingly, they tend to concentrate in regions with pervasive areas of replacement HGP (Palmieri et al., 2020). These findings emphasize the relevant interplay between the biology of CRCLM and how the HGP might define their TME. Additionally, LOXL4-expressing neutrophils might represent a possible biomarker for the replacement HGP (Palmieri et al., 2020).

As previously highlighted by van Dam et al. (2018), it is very likely that the immunologic TME and its tumor blood supply are conditionally related. As VEGF, the main mediator of sprouting angiogenesis, promotes not only angiogenesis, but also immunosuppression (Motz and Coukos, 2011), there might be an indication that the desmoplastic angiogenic HGP is related to an immunosuppressive TME, which is supported by the presence of tumor surrounding immune cells that are not penetrating the tumor (van Dam et al., 2018). Nevertheless, inflamed immune type tumors have been described in desmoplastic CRCLM (Stremitzer et al., 2020), alerting to controversial and ambiguous scenarios. Last, it is important to recognize that the hepatic sinusoidal endothelial cells, which surround the co-opted vessels in the liver, strongly contribute to tolerant immune responses (Knolle and Gerken, 2000; van Dam et al., 2018), rather than induction of immunity, as the constant contact with ingested and systemic circulating antigens could trigger severe and deleterious outcomes in the organ. Taken together, the immunologic status of the different HGP in CRCLM is still a controversial topic which requires further research.

### Potential Drivers for the Different HGP in CRCLM

To date, it is not yet elucidated which molecular drivers support CRCLM growth in different HGP (van Dam et al., 2017). One hypothesis consists in the assumption that the way how the liver reacts to damage has an impact on the expression of the different HGP (van Dam et al., 2017, 2018). This hypothesis points out to the interaction between tumor cells and their microenvironment. Both, desmoplastic CRCLM and liver fibrosis show ductular reactions (van Dam et al., 2018; Sato et al., 2019). Therefore, the desmoplastic HGP resembles a fibrotic reaction of the liver to damage (van Dam et al., 2018). In contrast to that, the replacement HGP shows some parallels with liver regeneration as reaction to liver damage, since the tumor cells replace the hepatocytes within the pre-existing liver cell plates. This resembles the physiological regeneration in the liver, where new hepatocytes also grow in the pre-existing plates without generating new ones (van Dam et al., 2018; Sato et al., 2019).

Another hypothesis emphasizes the expression of adhesion molecules, including L1CAM, β1-integrin, and α3-integrin as key players in VCO in several brain tumors (Dome et al., 2003; Carbonell et al., 2009; Bugyik et al., 2011; Valiente et al., 2014). These findings, deduced from brain tumors, could potentially also play a role in the vessel co-opting replacement HGP in CRCLM. Furthermore, tumor cell motility represents a crucial feature in VCO, as shown by Frentzas et al. (2016). The knockdown of the actin related protein complex 2/3 effectively inhibited VCO, suggesting that cell motility might play an essential role in the promotion of this mode of vascularization (Frentzas et al., 2016).

## Clinical Implications of Different HGP

The HGP and its TME composition are not only questions of academic interest, but they also have relevant clinical implications. In CRCLM, different HGP come along with different survival prognosis.

Generally, patients with desmoplastic CRCLM tend to have an improved overall survival (OS) when compared to other HGP (Nyström et al., 2012; Nielsen et al., 2014; Siriwardana et al., 2016; Galjart et al., 2019). Especially the pure desmoplastic HGP is associated with a favorable prognosis, whereas the presence of any amount of non-desmoplastic HGP impairs the prognosis (Galjart et al., 2019). Conversely, impaired OS was described for the replacement HGP in comparison to other HGP (Nielsen et al., 2014). Whilst the amount of desmoplastic HGP increases after the application of pre-operative chemotherapy, the survival benefit for patients with this HGP fades (Galjart et al., 2019). This has been shown by Galjart et al. (2019) in one analysis based on a cohort of patients with CRCLM. However, in a chemo-naive subgroup, pure desmoplastic HGP was significantly associated with improved OS and progression-free survival in multivariate analyses, in a pre-treated subgroup this survival benefit remained only significant in univariate analysis, but not in multivariate analysis (Galjart et al., 2019). Regarding the pushing HGP in CRCLM, different trends were described. While an intermediate survival between desmoplastic and replacement CRCLM was shown by Nielsen et al. (2014), other analyses pointed out to an association with a generally impaired OS (Falcao et al., 2018) or even identified pushing HGP as a prognostic marker for poor survival (Van den Eynden et al., 2012).

Additionally, the HGP can have clinical impact on disease progression after resection of the CRCLM. Firstly, when the resected CRCLM shows a desmoplastic HGP, the recurrence-free survival is improved (Eefsen et al., 2015), whereas the replacement HGP has been identified as an independent risk factor for both, intrahepatic and overall recurrences (Pinheiro et al., 2014).

Secondly, concerning local recurrences after CRCLM resection, patients with previously desmoplastic CRCLM tend to have generally a lower rate of recurrences than other HGP (Lunevicius et al., 2001). Moreover, in case of recurrence, patients with previously desmoplastic CRCLM tend to recur restricted to the liver, whilst patients, with previously non-desmoplastic CRCLM tend to recur more often in multiple locations and organs (Nierop et al., 2019).

Likewise, further studies investigated the prognostic value of HGP in LM derived from other primaries. In a cohort of patients with uveal melanoma LM, a predominant desmoplastic HGP was significantly associated with an improved OS compared to a predominant replacement HGP (Barnhill et al., 2018). Similar results were obtained from one analysis performed on patients with LM derived from cutaneous melanoma. Hereby, a predominantly desmoplastic HGP was also associated with a significantly improved OS compared to predominantly replacement HGP or mixed HGP (Barnhill et al., 2020). Moreover, this favorable prognostic effect of the desmoplastic HGP was even stronger when pure desmoplastic HGP were compared to any amount of replacement HGP (Barnhill et al., 2020), which is similar to the previously described results that Galjart et al. (2019) reported in a cohort of patients with CRCLM. In another analysis focused on HGP in LM derived from breast cancer, LM displaying any amount of desmoplastic HGP were associated with significantly improved progression free survival and OS when compared to LM with a pure replacement HGP (Bohlok et al., 2020). Last, recent analysis performed with patients diagnosed with pancreatic cancer LM showed that a predominant replacement HGP in these LM represents an independent factor for a poor prognosis (Watanabe et al., 2020). When compared to non-predominantly replacement HGP, the predominant replacement HGP was significantly associated with impaired OS (Watanabe et al., 2020). Limitations of this latter analysis might consist in the usage of LM-needle biopsies for scoring the HGP. However, by analyzing also excisional biopsies, the authors were able to show that most of the LM derived from pancreatic cancer express a homogenous HGP, which was defined as the expression of a specific HGP in >80% of the tumor-liver-interface (Watanabe et al., 2020). Thus, the usage of needle biopsies might represent an adequate method for HGP assessment in the specific context of prognostic relevance of HGP in pancreatic LM (Watanabe et al., 2020).

Taken together, these findings reveal that the different HGP are importantly related to the clinical outcome with a considerable impact on patients' further prognosis. Importantly, the prognostic impact and the clinical relevance of the HGP displayed by the LM was shown for different primary tumor entities.

### Anti-angiogenic Approach

Targeting sprouting angiogenesis in CRCLM is well-established since 2004, when the monoclonal antibody bevacizumab, which inhibits sprouting angiogenesis through VEGF inhibition, was approved by the FDA for usage in combination with chemotherapy in mCRC (Hurwitz et al., 2004). To date, the range of anti-angiogenic agents that are approved by the FDA for treatment of mCRC in specific settings is enlarged by further anti-angiogenic agents, such as aflibercept, ramucirumab, and regorafenib (Ciombor and Bekaii-Saab, 2018). Although all of the approved anti-angiogenic agents inhibit sprouting angiogenesis, they differ regarding their exact pharmacological mode of action, their usage in specific settings and combinations with or without chemotherapy and by their clinical effects on the patients' survival (Lopez et al., 2019). Bevacizumab was shown to effectively improve the OS of patients diagnosed with mCRC when combined with chemotherapy, compared to chemotherapy and placebo (median survival 20.3 vs. 15.6 months; Hurwitz et al., 2004). Aflibercept is a recombinant fusion protein that prevents VEGF binding its receptor with high affinity (Van Cutsem et al., 2012). Hence, it is also known as a VEGF Trap (Van Cutsem et al., 2012). In combination with chemotherapy, aflibercept also improves the OS of patients diagnosed with mCRC, when compared to chemotherapy and placebo (median survival 13.5 vs. 12.06 months; Van Cutsem et al., 2012). Ramucirumab is a monoclonal antibody, which specifically binds the extracellular domain of VEGF-receptor-2 preventing the binding of an endogenous ligand (Tabernero et al., 2015). In a second-line setting of patients diagnosed with mCRC, the application of ramucirumab in combination with chemotherapy showed an improved OS when compared to chemotherapy and placebo (median survival 13.3 vs. 11.7 months; Tabernero et al., 2015). The small molecule multi-kinase inhibitor regorafenib inhibits different kinases involved in tumor angiogenesis, kinases involved in oncogenesis, and kinases involved in signalling pathways in the TME (Grothey et al., 2013; Li et al., 2015). Its anti-angiogenic effect is based on the inhibition of VEGF-receptors-1–3 (Grothey et al., 2013; Li et al., 2015). In a cohort of patients with mCRC, who received at least two treatment lines before, the application of regorafenib resulted in improved OS when compared with the placebo group (median survival 8.8 vs. 6.3 months; Li et al., 2015). Also, in a multicenter based study of patients with mCRC that failed standard therapeutic regimes, the application of regorafenib compared to placebo prolonged the OS (median survival 6.4 vs. 5.0 months; Grothey et al., 2013). Altogether, anti-angiogenic treatment strategies represent an important and effective tool to improve the prognosis of patients diagnosed with mCRC in specific settings (Lopez et al., 2019).

Nevertheless, besides the described specific anti-angiogenic agents, other agents targeting the epidermal growth-factor are approved by FDA (Ciombor and Bekaii-Saab, 2018).

Thus, the mechanism of sprouting angiogenesis is already being targeted by several established therapeutic agents. However, the prolongation of the survival enabled by anti-angiogenic agents usually still remains limited to months or even weeks (Hurwitz et al., 2004; Van Cutsem et al., 2012; Grothey et al., 2013; Li et al., 2015; Tabernero et al., 2015). Unfortunately, other types of tumor vascularization than sprouting angiogenesis may still remain unaffected. Therefore, it is of crucial interest to investigate whether targeting other modes of tumor vascularization exclusively or additionally to anti-angiogenic treatment might have the potential to further improve patients' survival.

### Anti-VCO Approaches?

The fact that vessel co-opting CRCLM are not only less responsive to anti-angiogenic therapies than angiogenic CRCLM, but that they might also occur as a form of acquired resistance after the application of anti-angiogenic treatment, has been suggested by Frentzas et al. (2016). Generally, VCO represents an effective resistance mechanism to anti-angiogenic therapies not only in CRCLM but also in many different malignancies (Kuczynski and Reynolds, 2020). Therefore, VCO as an important tumor characteristic might be considered in the future when investigating or planning therapeutic strategies (Kuczynski and Reynolds, 2020). This highlights the necessity of an alternative therapeutic option for targeting vessel co-opting CRCLM, which to date remains unchallenged. As mentioned, in a LM mouse model, it has been demonstrated that the knockdown of the actin related protein complex 2/3 results in suppression of VCO, suggesting that cell motility represents a potential effective target (Frentzas et al., 2016). Other potential therapeutic targets, that were considered in other vessel co-opting tumor entities than CRCLM, affect cell adhesion, the expression of endothelial markers, the angiopoietin signalling and immune checkpoint inhibition (Kuczynski et al., 2019). Importantly, as VCO seems to define the tumor biology and its microenvironment in different tumor entities, new therapeutic approaches targeting the mechanism of VCO may not be limited to specific tumor types, but also represent a therapeutic option for several other vessel co-opting malignancies.

### Approaches to Assess the Histopathological Growth Pattern Prior to Resection

In order to achieve clinical benefit from the knowledge about the HGP and thus the biology of the CRCLM, it is paramount to find a reliable clinically available method to identify different HGP.

As Latacz et al. elaborated, several analyses have paved the way for medical imaging based in HGP assessment (Latacz et al., 2020b). Already two decades ago, the association of the thickness of the peritumoral border around LM was associated with enhanced perilesional gadolinium-uptake in magnetic resonance imaging (Semelka et al., 2000). However, limitations of this study include a small number of patients and varying primary tumors (Semelka et al., 2000).

In a retrospective study, the pre-operative identification of the predominant HGP of CRCLM was successfully performed by using a radiomics model, based on contrast-enhanced multidetector computer tomography (Cheng et al., 2019). Interestingly, an enhancement of the tumors rim was significantly stronger associated with the presence of a predominantly desmoplastic HGP (Cheng et al., 2019). Additionally, to the detection of the HGP in CRCLM (Cheng et al., 2019), also the response to treatment can be deduced from imaging (Chun et al., 2009). In a cohort of patients with CRCLM, who underwent a pre-treatment with bevacizumab and chemotherapy, a clear association of the morphological response in contrast-enhanced computer tomography scans with the pathological response to the treatment has been identified (Chun et al., 2009). The morphological response was assessed by rim enhancement, overall attenuation, and characteristics of the tumor liver interface (Chun et al., 2009). Recently, Han et al. (2020) showed that also a magnetic resonance based radiomics model is able to predict the predominant HGP in CRCLM. Especially radiomics of the tumor-liver-interface, combined with clinical characteristics provide a high diagnostic accuracy (Han et al., 2020). Thus, these findings indicate the possibility to deduce the pathological response of CRCLM from a non-invasive imaging method.

Besides imaging, another non-invasive strategy to achieve further information about tumor characteristics consists in the analysis of liquid biopsies (Poulet et al., 2019). In a cohort of patients, who received bevacizumab treatment, it was shown that several microRNAs found in extracellular vesicles, are potential predictive biomarkers for patients' prognosis (de Miguel Perez et al., 2020). However, in this study an association with histological aspects is lacking. An additional promising biomarker in liquid biopsy analysis is the expression of LOXL4 in circulating neutrophils, which seems to be higher in patients suffering from CRCLM than in healthy subjects (Palmieri et al., 2020). Nevertheless, a clear association with its expression and different HGP must be further characterized (Palmieri et al., 2020).

Furthermore, the measurement of collagen combined with the conventional measurement of the carcinoembryonic antigen (CEA) provides an improved non-invasive and non-imaging detection of CRCLM (Nyström et al., 2015; Lalmahomed et al., 2016; van Huizen et al., 2020). For instance, the assessment of both, circulatory collagen type IV and CEA in blood samples, results in a more sensitive detection of CRCLM (Nyström et al., 2015). Additionally, enhancement of both markers is related to impaired survival (Nyström et al., 2015). Although the assessment of collagen type IV improves the diagnostic of CRCLM, a relationship of the preoperative collagen type IV levels with the expression of specific HGP in the CRCLM was not found (Nyström et al., 2015). Besides blood analysis, also urine markers have the potential to effectively contribute to the detection of CRCLM (Lalmahomed et al., 2016; van Huizen et al., 2020). The combination of the CEA level in serum combined with specific collagen markers in urine provide a detection of CRCLM with a high diagnostic accuracy (Lalmahomed et al., 2016; van Huizen et al., 2020). However, hereby the expressed HGP in the diagnosed CRCLM was not investigated (Lalmahomed et al., 2016; van Huizen et al., 2020).

## Role of Vessel Co-Option in Different Tumor Entities

### Vessel Co-option in Lung Tumors

Like many other tumors, the development and metastasis of lung tumors requires adequate nutrients supplied by blood vessels. For many years, angiogenesis has been considered to be crucial for tumor formation (Hanahan and Folkman, 1996; Kuczynski et al., 2019). A variety of targeted drugs, are clinically used for targeting angiogenesis, including the VEGF antibody bevacizumab (Sandler et al., 2006), the VEGF-recepotor-2 antibody ramucirumab (Garon et al., 2014), and nintedanib (Reck et al., 2014), a tyrosine kinase inhibitor with activity against VEGF-receptors (Janning and Loges, 2018). However, the clinical benefit from anti-angiogenic treatment of lung tumors is not yet as satisfactory as expected (Janning and Loges, 2018). In 1996, Pezzella et al. reported the occurrence of VCO in several primary and secondary lung tumors (Pezzella et al., 1996, 1997). This finding led to the hypothesis that the angiogenesis-independent growth might affect the efficacy of anti-angiogenic treatment strategies (Pezzella et al., 1997). In this section, we discuss specific examples of VCO in primary and secondary lung tumors and consider the clinical significance of this alternative tumor blood supply.

#### Primary Lung Cancer

**Figure 4:**
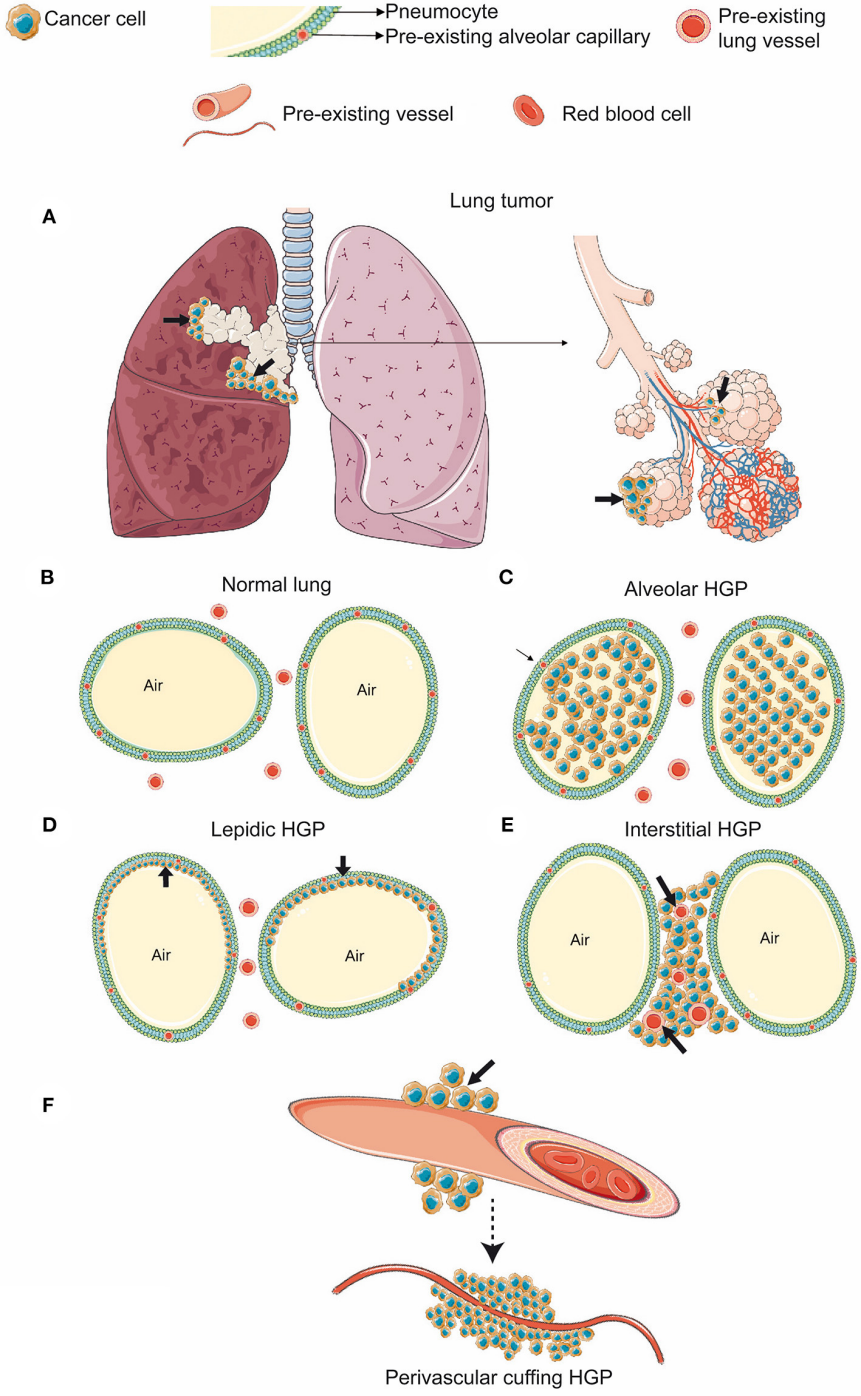
HGP in primary lung cancer **(A–F)**. **(A)** Cancer cells growing in the bronchus of the lung (black arrows). **(B)** In the normal alveolar parenchyma, the lung alveolar spaces are filled by air and are delimited by the alveolar walls. **(C)** Alveolar HGP. Cancer cells fill the alveolar air space. **(D)** Lepidic HGP. Cancer cells spread along the alveolar walls, but preserve some air space by not filling the alveoli completely. **(E)** Interstitial HGP. Cancer cells grow between the alveoli, but do not enter the alveolar space. **(F)** Perivascular cuffing HGP. Cancer cells grow cuff-like around blood vessels. (Figure created with vectors from Servier Medical Art. https://smart.servier.com/).

Pezzella et al. (1996) proposed that in the lung, which provides an optimal vascular bed around empty spaces Figures 4A,B primary lung cancers and metastases to the lungs could develop without producing either new vessels or any tumor stroma. Additionally, they observed that primary lung tumors, using local blood vessels of normal lung tissue, tend to be more aggressive (Pezzella et al., 1996). Generally, different HGP can be distinguished in primary lung tumors (Kuczynski et al., 2019). In 1997, Pezzella et al. described four different tumor HGP in the lung (Pezzella et al., 1997). The (i) papillary, the (ii) basal, and the (iii) diffuse HGP are characterized by destroying the normal lung tissue and the interstitium and are accompanied by newly formed blood vessels (Pezzella et al., 1997). Conversely, the (iv) alveolar HGP, growing within the alveoli (Figure 4C), uses VCO for ensuring its blood supply (Pezzella et al., 1997), respects the structure of the pre-existing alveoli, and grows without destroying the lung parenchyma (Pezzella et al., 1997). Yet, another classification, suggested by Sardari Nia et al. (2008), distinguishes between three different HGP with respect to their mechanism of blood supply. Hereby, the (i) destructive HGP, which resembles the previously mentioned basal and diffuse HGP (Kuczynski et al., 2019), is characterized by angiogenic growth, the (ii) papillary HGP is known for using both, angiogenic and vessel co-opting growth, whereas the (iii) alveolar HGP is defined by angiogenesis-independent growth applying VCO (Sardari Nia et al., 2008; Figure 4C). Generally, the vessel co-opting alveolar HGP is characterized by cancer cells, filling the air gap of the alveoli and co-opting the pre-existing vessels (Pezzella et al., 1997; Sardari Nia et al., 2008; Donnem et al., 2018; Figure 4C). Additionally, another established HGP of primary lung cancer consists in the lepidic HGP (Travis et al., 2011; Kuczynski et al., 2019; Figure 4D). Hereby, the cancer cells spread along preexisting alveolar walls (Travis et al., 2011; Kuczynski et al., 2019; Nakamura et al., 2020), which allows them to co-opt alveolar capillaries (Kuczynski et al., 2019). In contrast to the alveolar HGP, the lepidic HGP is characterized by preserving some space within the alveoli, instead of completely filling the airspace (Kuczynski et al., 2019). In 2015, the World Health Organization added a new specific invasive pattern of lung tumors, named “spread through air spaces” characterized by cancer cells invading the alveoli (Travis et al., 2015). Recently, it has been shown that in this scenario, tumor cells co-opt pre-existing capillaries while attaching to the alveolar walls (Yagi et al., 2020). Additionally, researchers have shown that this newly defined pattern of lung cancer invasion was associated with aggressive clinical pathologic factors (Kadota et al., 2015; Lu et al., 2017).

#### Lung Metastases

In addition to primary lung tumors, also in metastatic pulmonary lesions, different HGP are associated with either angiogenic or vessel co-opting blood supply (Bridgeman et al., 2017; Kuczynski et al., 2019). In one of the characterized HGP, called pushing or destructive HGP, the blood supply is obtained via angiogenesis. In contrast to that, four other HGP are characterized by obtaining their blood supply through VCO. The latter ones include the (i) alveolar HGP (Figure 4C), the (ii) lepidic HGP (Figure 4D), the (iii) interstitial HGP (Figure 4E), and the (iv) perivascular cuffing HGP (Figure 4F) (Kuczynski et al., 2019). The evaluation of HGP in human lung metastases, derived from colorectal, renal, and breast cancer primaries confirmed the regular occurrence of VCO in their lung metastases (Bridgeman et al., 2017). Interestingly, it must be acknowledged that eventually both VCO and angiogenesis may occur inside the same metastatic lesion (Bridgeman et al., 2017). It was further hypothesized that in case of alveolar and interstitial HGP, it is conceivable that newly sprouting vessels can originate from co-opted ones as long as the co-opted vessels are located in the center of the metastatic lesion (Bridgeman et al., 2017). Nevertheless, new studies are still necessary to elucidate which are the molecular signals involved in these vascular-cancer cell interactions and which are the factors determining vascular blood supply patterns.

### Vessel Co-option in Brain Tumors

Likewise, in brain tumors VCO is a relevant mechanism of blood supply besides angiogenesis (Seano and Jain, 2020). The occurrence of brain tumors, growing independently from angiogenesis has been described already long ago (Holash et al., 1999). In the following, we will discuss primary and secondary brain tumors in the context of VCO.

#### Primary Glioma and Their Vascularization

**Figure 5:**
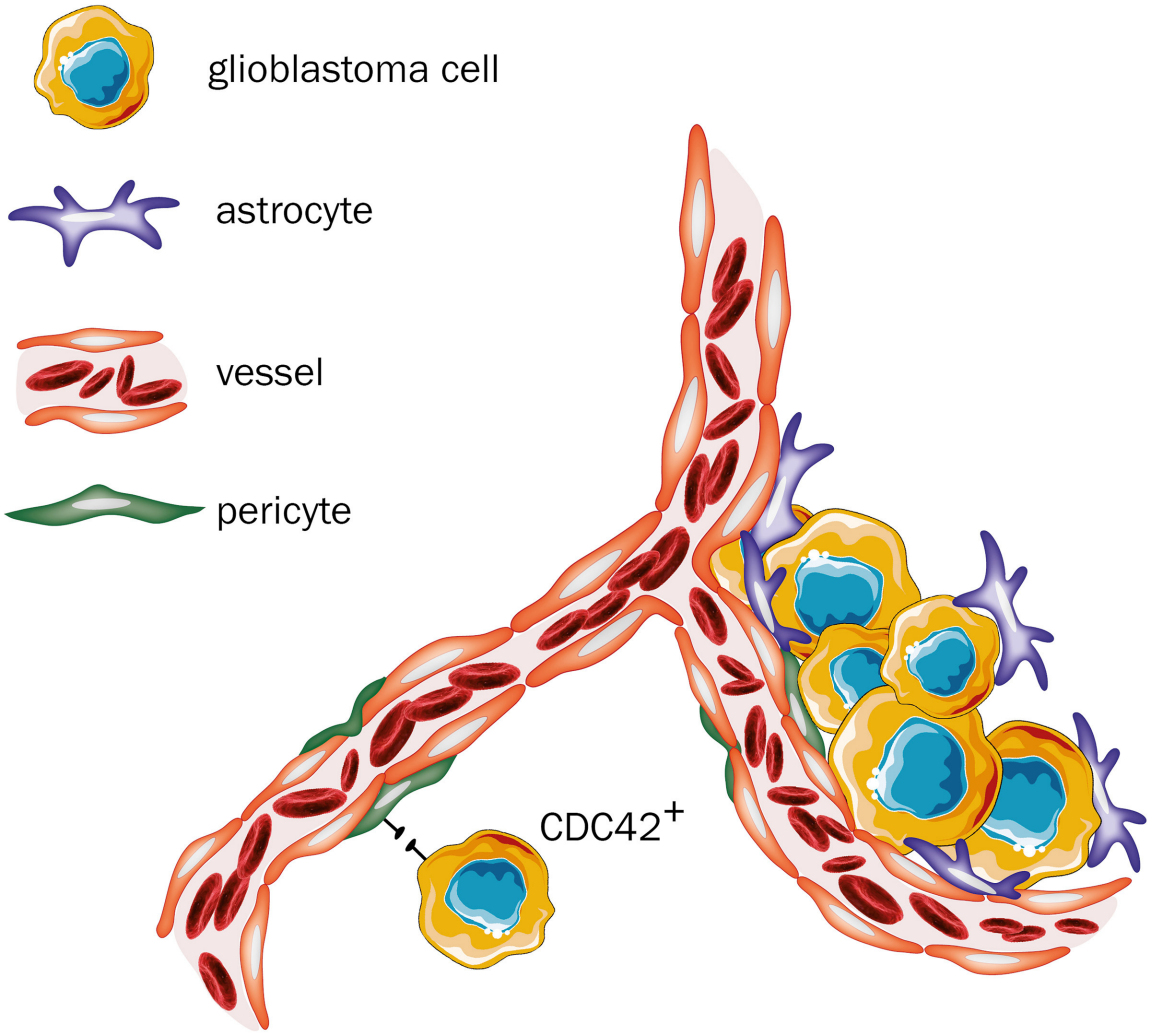
Glioblastoma—primary tumor. Once cancer cells reach the blood vessels, two outcomes are feasible: (i) neoplastic cells produce flectopodia and fuse with pericytes through CDC42-mediated processes to produce hybrid cell types. However, when CDC42 activity is blocked, fusion is abrogated, and pericytes might gain anti-tumor activities (assuming a peripheral-derived macrophage-like phenotype). (ii) tumor cells place themselves between astrocytes and pericytes, thereby blocking their physiological interactions and changing their normal functions (Figure created with vectors from Servier Medical Art. https://smart.servier.com/).

Glioblastoma is the most common malignant tumor in the brain (Ostrom et al., 2018) and although this tumor entity rarely forms distant metastases, it is one of the most invasive and aggressive intracranial malignancies (Agnihotri et al., 2013). It has been already shown that besides angiogenesis, VCO is a relevant mechanism of blood supply in glioblastoma (Seano and Jain, 2020) and that glioblastoma cells can grow independently from angiogenesis (Holash et al., 1999; Seano and Jain, 2020). Previously, an *in vivo* rat model, showed that anti-VEGF treatment leads to slower growth of intracranial glioblastoma tumors, however their histological evaluation revealed an increased invasive tumor growth and co-option of pre-existing vessels, indicating acquired strong resistance mechanisms to anti-angiogenic therapy (Rubenstein et al., 2000). Additionally, in an angiogenic xenograft mouse model, bearing glioblastoma cells, treatment with the anti-angiogenic agent bevacizumab resulted in promoted tumor cell infiltration, suggesting that VCO could represent an escape mechanism from anti-angiogenic treatment (de Groot et al., 2010). Moreover, analysis performed with human brain tissue from autopsies further elucidated the impact of anti-angiogenic treatment on the vascular structure of recurrent glioblastoma (di Tomaso et al., 2011). Subjects who received cediranib, a pan-VEGF-receptor tyrosine kinase inhibitor, showed a vessel structure more similar to the tumor-free brain tissues without indication of further angiogenesis, when compared to the subgroup who did not receive anti-angiogenic therapy (di Tomaso et al., 2011). Therefore, this switch in growth might represent a clinically relevant acquired resistance mechanism following anti-angiogenic therapy (di Tomaso et al., 2011), which potentially involves VCO (Kuczynski et al., 2019). Further evidence for VCO acting as a resistance mechanism to anti-angiogenic treatment was found in one analysis based on tumor tissue from three patients, who were diagnosed with recurrent glioma despite anti-angiogenic therapy with bevacizumab, and on a murine model for glioma also treated with bevacizumab (de Groot et al., 2010). In this analysis, histological examination of the tissue obtained from the previously described three patients revealed tumor vessel normalization without evidence for perivascular invasion, whereas in the murine tissue samples, perivascular invasion was clearly present, indicating that VCO may be involved in clinically relevant resistance mechanisms to anti-angiogenic therapy in recurrent glioma (de Groot et al., 2010). At the cellular and molecular levels, some new insights on glioblastoma and its vascular nature have already been reported. Caspani et al. (2014) precisely evaluated the steps of VCO in glioblastoma, demonstrating that once cancer cells reach the blood vessels, they produce flectopodia, which can alter the contractility of the pericytes. Moreover, the authors have also shown that cancer cells can fuse with pericytes, a process mediated by CDC42, to create new hybrid cell types (Caspani et al., 2014; Figure 5). Also, Watkins et al. (2014) described in detail that glioblastoma cells place themselves under astrocytic end-feet interrupting coupling of astrocytes to blood vessels (Figure 5). Recently, new molecular pieces of evidence have been reported on glioblastomas and VCO interactions. Griveau et al. (2018) showed that WNT7-induced Oligo2^+^-oligodendrocyte precursor-like cells are able to invade the brain parenchyma in a VCO-manner without disrupting the adjacent vasculature. Crucially, they observed that Oligo2^−^-cancer cells grow as perivascular clusters, directly affecting the blood brain barrier and triggering an immune cell activation (Griveau et al., 2018). These findings strongly support the idea that specific molecular traits are involved in tumors vascular choices. Moreover, they suggested that WNT signalling might be directly involved in this process.

#### Brain Metastases

**Figure 6:**
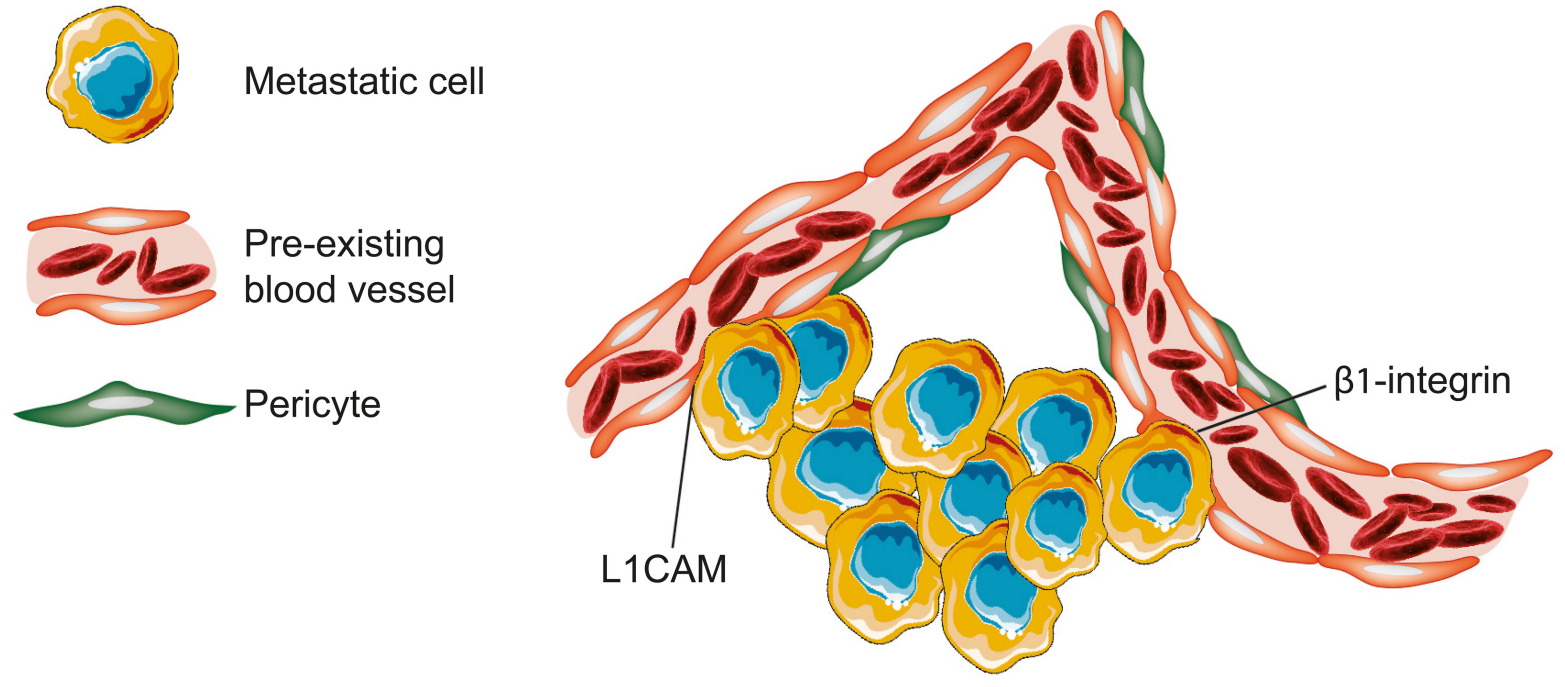
Brain—metastases. In brain metastases, tumor cells can attach to the vascular basement membrane via β1-integrin. The resultant close spatial proximity to vessels allows then the metastatic cells to co-opt the pre-existing blood vessel. In the presented model, non-angiogenic metastatic cells infiltrating the brain tissue are characterized by secretion of neuroserine protease inhibitors and expression of L1CAM. Neuroserpin blocks plasminogen activator and prevents the formation of plasmin. The inhibition of plasmin release into the microenvironment prevents both the death of cancer cells by FAS ligand and the further blockade of L1CAM (Figure created with vectors from Servier Medical Art. https://smart.servier.com/).

Investigations in the field of brain metastases based on analysis of human tissue samples and different *in vivo* models revealed that brain metastases use VCO to achieve blood supply (Kienast et al., 2010; Bugyik et al., 2011; Berghoff et al., 2013; Siam et al., 2015; Bentolila et al., 2016). In case of VCO in brain metastases, it can be understood as an alternative to neovascularization, and probably plays a crucial role in transporting nutrients and oxygen (Carbonell et al., 2009). In a murine model of brain metastases, it was shown that the expression of the adhesion molecule β1-integrin by cancer cells is required for an adequate adhesion to the vascular basement membrane of vessels (Carbonell et al., 2009). This molecular connection allows the cancer cells to co-opt the particular vessel (Carbonell et al., 2009). In 2014, Valiente et al. proposed a mechanism for VCO in brain metastases (Valiente et al., 2014). In that, one important driver for VCO in brain metastasis is the adhesion molecule L1CAM which allows the metastatic cells to spread on the capillaries and to co-opt them (Valiente et al., 2014). However, the enzyme plasmin has the power to inhibit this metastatic process, as it is involved in the inactivation of L1CAM and in the mobilization of FAS ligand (Valiente et al., 2014). By secreting plasminogen activator inhibitory serpins, brain metastatic cells prevent the formation of plasmin (Valiente et al., 2014). Thus, the expression of plasminogen activator inhibitory serpins allows the metastatic cells to continue benefiting from L1CAM spreading along capillaries and avoid FAS induced cell death mediated by plasmin (Valiente et al., 2014; Figure 6).

### Vessel Co-option at Other Tumor Sites Than Lung and Brain

Vessel co-option is also known to occur in other tissues, including tumors in the skin and in lymph nodes (Naresh et al., 2001; Dome et al., 2002). Melanoma tumor cells of the skin fuse with the surrounding normal blood vessels to obtain nutrients and support their own growth and survival (Dome et al., 2002). Lymph nodes have a dense network of blood vessels, which can provide ideal nutrition for tumor cells, so lymph node metastases are able to exploit preexisting vessels (Naresh et al., 2001). The currently clinically approved angiogenesis inhibitors are not active during early cancer progression in the lymph node, suggesting that inhibitors of sprouting angiogenesis will not be able to treat or to prevent lymph node metastases effectively (Jeong et al., 2015). However, lymph node tumors do not grow completely independent from sprouting angiogenesis (Vermeulen et al., 2002). Instead of that, lymph node tumors seem to grow in different grades of angiogenesis-independency depending on their actual growth patterns (Vermeulen et al., 2002). Renal cell carcinomas also use VCO, which might be driven by an epithelial-to-mesenchymal-like invasive process (Bridgeman et al., 2017).

## Discussion

The widely varying diversity of the TME is a topic of current research. As the different types of blood supply have clear clinical implications, an improved understanding is urgently needed. Not only in CRCLM, but also in many other tumor entities, VCO was identified as an underlying mechanism of blood supply that is often associated with a specific morphology and a poor prognosis (van Dam et al., 2017).

Although the inhibition of sprouting angiogenesis in CRCLM is for more than a dozen years a well-established therapeutic anti-cancer strategy (Hurwitz et al., 2004), to date there is no equivalent approach for targeting VCO (Kuczynski et al., 2019). Moreover, the occurrence of the vessel co-opting replacement HGP in CRCLM shows a considerable association with impaired survival (Fernandez Moro et al., 2018), indicating that VCO and TME play a crucial role in disease progression. As Frentzas et al. (2016) showed, replacement HGP tend to occur more frequently after anti-angiogenic treatment. This could implicate that VCO might represent a mode of acquired resistance to anti-angiogenic approaches. Consequently, targeting VCO could also prolong patients' survival. Another potential strategy in vessel co-opting CRCLM might consist in targeting cancer cell motility (Frentzas et al., 2016).

Stremitzer et al. (2020) showed a considerable association between the immunologic TME and patient survival. An inflamed TME in CRCLM is associated with improved recurrence-free survival and shows a trend for an improved OS after resection (Stremitzer et al., 2020). In contrast to that, immune cell infiltration is nearly not existing at all in replacement HGP (van Dam et al., 2017) and this HGP could even be described as a desert immune phenotype (van Dam et al., 2018). Thus, promotion of immune cell infiltration in replacement CRCLM could also result in an improved survival.

Interestingly, the desert immune phenotype observed in replacement HGP (van Dam et al., 2018) shows a higher level of LOXL4-positive neutrophils than desmoplastic HGP (Palmieri et al., 2020), which emphasizes the role of rare immune cells in the replacement HGP. Nevertheless, also in the desmoplastic HGP LOXL4-positive neutrophils were observed, but they were mainly concentrated on areas, where replacement HGP pervades and might indicate the conversion of desmoplastic to replacement HGP (Palmieri et al., 2020). Taken these results together, it would be interesting to investigate whether LOXL4 expression plays a role in acquired resistance.

Besides therapeutic challenges, a pre-operative diagnosis of the exact HGP in CRCLM is still an essential topic of research to achieve the maximum clinical benefit for CRCLM patients. As previously described, multi-detector computer tomography, and also radiation-free magnet resonance based radiomics seem to be promising tools to assess the predominant HGP (Cheng et al., 2019; Han et al., 2020). However, in both techniques the predominant HGP was classified as such, when >50% of the tumor border displayed the specific HGP (Cheng et al., 2019; Han et al., 2020). Referring to the study of Galjart et al. (2019), the presence of any non-desmoplastic HGP in CRCLM impairs the patients' prognosis. Therefore, further research on imaging methods is required to achieve an optimized diagnostic accuracy with a precise prediction of the HGP. Fortunately, new findings about imaging methods combined with a detailed analysis of the HGP in CRCLM can be expected, as a prospective study, addressing this issue, is currently ongoing (Latacz et al., 2020b).

As enhanced imaging contrast in the portal venous phase seems to be a reliable characteristic of the HGP in computer tomography based radiomics (Cheng et al., 2019), the analysis of enhancement characteristics in ultrasound images could represent another potential method for rough prediction of HGP in CRCLM with a radiation-free and easily accessible method. However, also in this scenario, a rough estimation might be insufficient, since the presence of any non-desmoplastic growth seems to affect the patients' prognosis, as previously described (Galjart et al., 2019).

Further exploration of liquid biopsy data might represent a promising perspective for HGP diagnosis and guidance for further therapeutical decisions (de Miguel Perez et al., 2020; Palmieri et al., 2020). Yet, the extension of the predictive value of this new method needs to be closer investigated.

Co-opting sinusoidal vessels directly implicate the access to blood enriched with nutrients. Consequently, co-opting CRCLM cells might be pre-disposed to altered metabolic states. Thus, it is well-conceivable that these cells might be prone to a metabolic switch toward glycolysis. The Warburg's effect is known for increased glucose uptake and increased lactate production, despite of sufficient oxygen supply, which leads to the promotion of cancer development (Liberti and Locasale, 2016). Both, the facilitated access to glucose, while co-opting the nutrient enriched sinusoids, as well as the decreased survival of patients with replacement CRCLM, could indicate a metabolic switch in direction of the Warburg's effect. Consequently, this would open a new field of therapeutic approaches for replacement CRCLM by targeting the cancer cell metabolism, a domain which absolutely has strong therapeutic potential (Luengo et al., 2017). Unfortunately, the metabolic status of CRCLM with differing HGP still remains to be accurately evaluated.

Considering the mechanisms of PM and the associated histopathological marker angiotropism, it would be highly interesting to elucidate further its relationship with VCO. Since in case of angiotropism tumor cells are localized on the abluminal side of vessels in a pericyte location (Barnhill and Lugassy, 2004; Lugassy et al., 2020), this spatial localization consecutively blends PM to VCO (Lugassy et al., 2020). Additionally, based on a mouse model, the close relationship of PM and VCO was described (Bentolila et al., 2016). However, angiotropism also subsumes a completely extraluminal migratory process along vessels (Lugassy et al., 2020), whereas in case of vessel co-opting metastases, one might simply assume an intravasal way of metastasis formation (Bentolila et al., 2016). However, the mechanism of angiotropism or PM should be considered as an alternative way of metastasis formation (Bentolila et al., 2016). Thus, additional analysis with a focus on the exact mechanisms of metastasis formation are required to elucidate whether metastases that are known to obtain their blood via VCO, arise from an extraluminal migration process or via the commonly expected intravasal dissemination process.

To summarize, TME and VCO play a key role in the biology of many different tumors. A direct association between the TME and clinical aspects has been clearly shown by several studies. Researchers have been able to point out important attributes of VCO in different tumor entities. However, the many underlying mechanisms are not fully elucidated. Thus, an extended understanding of VCO is absolutely required to improve not only the therapeutic strategies for patients with CRCLM, but also for patients suffering from other solid malignancies.

## Author Contributions

GH and SF drafted the manuscript. TD and L-CC conceptualized the review article. TD, SF, and GH designed the figures. All authors contributed to the article and approved the submitted version.

## Conflict of Interest

The authors declare that the research was conducted in the absence of any commercial or financial relationships that could be construed as a potential conflict of interest. The handling editor declared a past co-authorship with one of the authors L-CC.
